# T-Cell Receptor/CD3 Downregulation and Impaired Signaling in HTLV-1-Infected CD4+ T Cells of HAM Patients

**DOI:** 10.3390/ijms26041706

**Published:** 2025-02-17

**Authors:** Satoshi Nozuma, Toshio Matsuzaki, Masakazu Tanaka, Daisuke Kodama, Mika Dozono, Takashi Yoshida, Hiroshi Takashima, Ryuji Kubota

**Affiliations:** 1 Department of Neurology and Geriatrics, Graduate School of Medical and Dental Sciences, Kagoshima University, 8-35-1 Sakuragaoka, Kagoshima 890-8520, Japan; snozuma@m2.kufm.kagoshima-u.ac.jp (S.N.); k3783253@kadai.jp (M.D.);; 2 Division of Neuroimmunology, Joint Research Center for Human Retrovirus Infection, Kagoshima University, 8-35-1 Sakuragaoka, Kagoshima 890-8544, Japantanakam@m.kufm.kagoshima-u.ac.jp (M.T.); kodamada@m.kufm.kagoshima-u.ac.jp (D.K.)

**Keywords:** HTLV-1, HTLV-1-associated myelopathy, T-cell receptor, CD3, immune dysfunction

## Abstract

Human T-cell leukemia virus type 1 (HTLV-1) is a retrovirus associated with adult T-cell leukemia (ATL), a hematological malignancy, and HTLV-1-associated myelopathy (HAM), a progressive neurological disorder. HTLV-1 predominantly infects CD4+ T cells in vivo. The T-cell receptor (TCR)/CD3 complex on CD4+ helper T cells plays a pivotal role in immune responses by recognizing antigens and facilitating coordination with other immune cells. Dysfunction of the TCR/CD3 complex may impair immune function. Although CD3 downregulation has been identified as a characteristic of ATL cells, it remains uncertain whether a similar downregulation occurs in HTLV-1-infected cells from HAM patients. We hypothesized that HTLV-1 infection leads to TCR and CD3 downregulation, contributing to immune dysfunction in HAM patients. To test this hypothesis, we analyzed TCR/CD3 expression, TCR signaling, and immune responses in HTLV-1-infected cells from HAM patients. Intracellular HTLV-1 Tax detection revealed that HTLV-1 preferentially targets CD4+ over CD8+ T cells. CD3 and TCR expression levels were significantly lower in CD4+ T cells from HAM patients compared to healthy controls. Furthermore, HTLV-1-infected cells exhibited markedly reduced CD3 and TCR expression compared to uninfected cells. Impairments in TCR signaling, assessed through Lck and ZAP70 phosphorylation upon CD3 stimulation, were observed in CD4+ T cells from HAM patients compared to those from healthy controls. Notably, this reduction in TCR signaling was more pronounced in HTLV-1-infected CD4+ T cells than in uninfected CD4+ T cells in HAM patients. Additionally, cytomegalovirus (CMV)-specific CD4+ T cells detected by an addition of CMV antigens demonstrated reduced interferon-γ production in HTLV-1-infected cells compared to their uninfected counterparts. These findings suggest that TCR/CD3 downregulation and impaired TCR signaling contribute to immune dysfunction in HTLV-1-infected CD4+ T cells. As CD4+ T cells play a central role in immune responses, this mechanism may partially explain the cellular immune dysfunction to other pathogens observed in HAM patients.

## 1. Introduction

Human T-cell leukemia virus type 1 (HTLV-1) is a retrovirus that primarily infects CD4+ T cells in vivo, establishing a lifelong infection [1]. An estimated 10–20 million individuals worldwide are infected with HTLV-1, with endemic areas such as Japan reporting higher prevalence rates [2]. While most infected individuals remain asymptomatic, approximately 5% develop severe diseases, including adult T-cell leukemia (ATL), a hematological malignancy, or HTLV-1-associated myelopathy (HAM), a chronic inflammatory neurological disorder [3,4,5]. HAM primarily affects the spinal cord, manifesting as spastic paraparesis, sensory disturbances in the lower extremities, and sphincter dysfunction [6]. Patients with HAM typically exhibit higher HTLV-1 proviral loads, a key risk factor for disease progression, and elevated HTLV-1-specific cellular immune responses compared to asymptomatic carriers [7,8,9]. These heightened immune responses have sparked debate regarding the efficacy of HTLV-1-specific cytotoxic T lymphocytes (CTLs) in controlling infection, raising questions about whether these responses are excessively strong or paradoxically insufficient.

ATL is frequently complicated by opportunistic infections, such as bacterial and fungal infections, attributed to immunosuppression [10,11]. Similarly, asymptomatic HTLV-1 carriers demonstrate immune dysfunction, predisposing them to opportunistic infections [12,13,14]. In contrast, HAM, caused by HTLV-1-associated neuroinflammation rather than malignancy, has distinct immunological characteristics. Although HAM patients exhibit robust HTLV-1-specific immune responses, they demonstrate reduced skin reactivity to dinitrochlorobenzene (DNCB) and PPD-tuberculin, as well as diminished lymphoproliferative responses to mitogens such as phytohemagglutinin and pokeweed mitogen [15,16]. These observations suggest impaired immune responses to non-HTLV-1 pathogens in HAM patients. However, the underlying mechanisms of immune dysfunction in HAM patients remain poorly understood.

The T-cell receptor (TCR) plays a pivotal role in antigen recognition and the elimination of pathogens. TCRs are classified into two forms: TCRαβ and TCRγδ. The TCRαβ consists of a Fab-like clonotypic αβ heterodimer, which is noncovalently associated with an invariant, multimeric CD3 complex. This complex includes a CD3εδ heterodimer, a CD3εγ heterodimer, and a CD3ζζ homodimer. The invariant CD3 components are crucial for regulating the surface expression of the TCRαβ chains and facilitating signal transduction upon the binding of peptide–MHC ligands to the receptor’s variable domains [17]. Antigen binding to the TCR triggers signaling cascades involving key molecules such as Lck and ZAP70, ultimately leading to T-cell activation [18,19]. CD4+ T cells, functioning as helper T cells, play a crucial role in coordinating immune responses through interactions with other immune cells; their dysfunction can result in immunosuppression. For example, HIV-1 specifically targets CD4+ T cells, inducing cell death and leading to acquired immunodeficiency syndrome, which is characterized by recurrent opportunistic infections and malignancies [20]. Similarly, HTLV-1 predominantly infects CD4+ T cells, altering their function primarily through the viral protein Tax rather than causing cell death [21]. While CD3 downregulation has been documented on the surface of ATL cells, it remains uncertain whether a similar downregulation occurs in HTLV-1-infected cells from asymptomatic HTLV-1 carriers or HAM patients [22]. Furthermore, the immune functionality of HTLV-1-infected cells is not yet fully understood.

We hypothesized that HTLV-1 infection leads to TCR and CD3 downregulation, contributing to immune dysfunction in asymptomatic carriers and HAM patients. To test this hypothesis, we investigated TCR/CD3 expression, TCR signaling, and immune responses in HTLV-1-infected cells from HAM patients. Our findings revealed TCR/CD3 downregulation and impaired TCR signaling in HTLV-1-infected CD4+ T cells in HAM patients. Furthermore, these cells exhibited reduced interferon (IFN)-γ production against other viral antigens, suggesting impaired cellular immune responses to non-HTLV-1 pathogens.

## 2. Results

### 2.1. Detection of HTLV-1-Infected Cells in CD4+ and CD8+ Cells of HAM Patients

**Figure 1:**
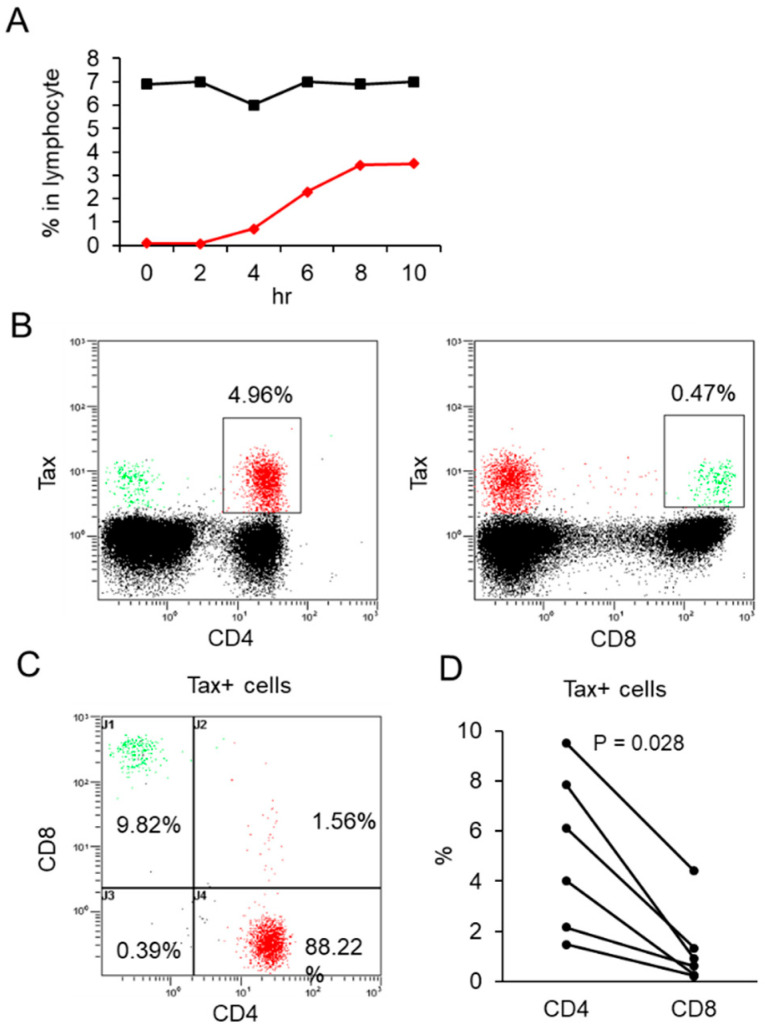
Detection of HTLV-1-infected cells in CD4+ and CD8+ cells of HAM patients. (**A**) Time-course analysis of HTLV-1-infected cells in culture without stimulation. The black and red curves represent the HTLV-1 proviral load and intracellular Tax+ cells, respectively. Tax staining detected 49.9% of infected cells. (**B**) Representative flow cytometry plots showing HTLV-1-infected cells within CD4+ T cells (red) and CD8+ T cells (green). (**C**) Proportions of Tax+ cells are analyzed based on CD4 and CD8 expressions. Among Tax+ cells, 88.22% are CD4+ (red, lower right quadrant), 9.82% are CD8+ (green, upper left quadrant), and 1.56% are CD4+CD8+ double-positive (red, upper right quadrant). Across six HAM patients, the mean ± SD proportions of infected cells are 81.4 ± 4.3% in CD4+ cells and 12.5 ± 8.5% in CD8+ cells. (**D**) Percentages of Tax+ cells within CD4+ and CD8+ cell populations. Quantitative analysis reveals 5.19 ± 3.19% of CD4+ cells and 1.30 ± 1.58% of CD8+ cells are Tax-positive.

A time-course study was conducted to optimize the detection of HTLV-1-infected cells. Intracellular HTLV-1 Tax-positive cells were first detected after 2 h of culture, reaching a peak at 8 h (Figure 1A). Notably, the HTLV-1 proviral load remained stable throughout the incubation period. Intracellular Tax detection identified 49.9% of HTLV-1-infected cells, as determined by proviral load quantification. Preliminary experiments demonstrated that intracellular Tax detection was more sensitive than surface CADM1 detection for identifying HTLV-1-infected cells. Based on these findings, subsequent analyses employed intracellular Tax detection following an 8-h incubation period. HTLV-1-infected cells were detected in both CD4+ and CD8+ T cell populations of HAM patients, as indicated by HTLV-1 Tax protein expression (Figure 1B,C). No Tax-positive cells were observed in peripheral blood mononuclear cells (PBMCs) from HAM patients stained with an isotype control antibody or from non-infected individuals stained with the Lt-4 antibody. The CD4+ and CD8+Tax+ cells exhibited high CD4 and CD8 expression, suggesting their identity as T cells. The majority of HTLV-1-infected cells were CD4+ T cells (88.22%), with smaller proportions comprising CD8+ T cells (9.82%) and CD4+CD8+ double-positive cells (1.56%) (Figure 1C). Among six HAM patients, 81.4 ± 4.3% (mean ± SD) of infected cells were CD4+, compared to 12.5 ± 8.5% that were CD8+, demonstrating a significantly higher frequency of infection in CD4+ T cells. Quantitative analysis further revealed that the proportion of infected cells was 5.19 ± 3.19% in CD4+ T cells versus 1.30 ± 1.58% in CD8+ T cells, indicating that CD4+ T cells were four times more likely to harbor HTLV-1 infection (Figure 1D). Phenotypic analysis showed that HTLV-1-infected cells exhibited reduced expression of CD7, CD26, CD45RA, and CCR7 (Supplemental Figure S1). Notably, 95.9% of infected cells were CD45RA-negative and CCR7-negative, indicative of an effector memory phenotype (Supplemental Figure S2A,B). Additionally, 71.5% of infected cells were CD28-positive and CD27-negative, reflecting an intermediate differentiation state (Supplemental Figure S2C,D). Furthermore, 40.6% of CD4+Tax+ cells were CCR4-positive, and intracellular FoxP3 analysis revealed a higher proportion of FoxP3-positive cells among CD4+Tax+ cells (2.70 ± 2.43%) compared to CD4+Tax− cells (1.27 ± 0.60%).

### 2.2. Downregulation of TCR/CD3 in HTLV-1-Infected Cells of HAM Patients

**Figure 2:**
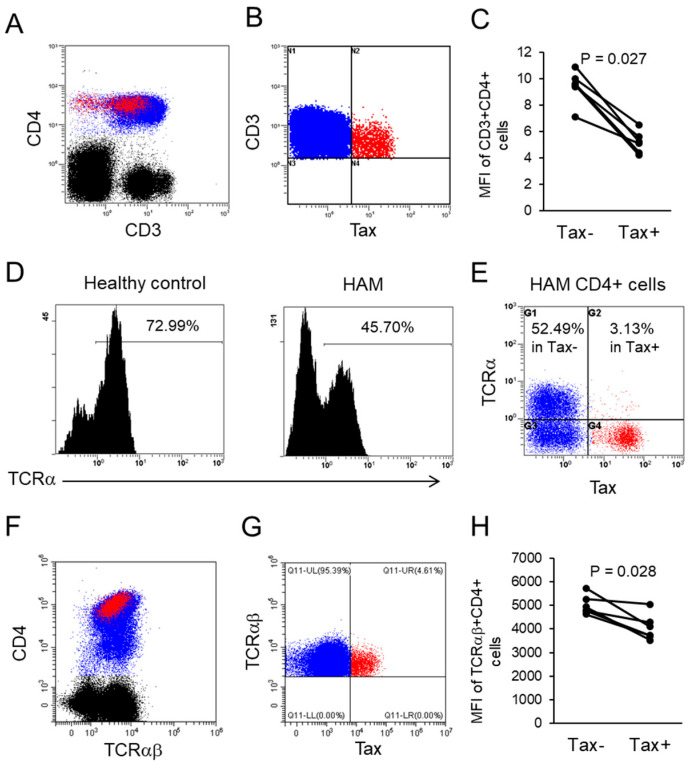
Downregulation of TCR/CD3 in HTLV-1-infected cells of HAM patients. (**A**) Representative flow cytometry plots illustrating CD3 downregulation in CD4+ HTLV-1-infected cells. Tax-positive and Tax-negative CD4+ cells are shown in red and blue, respectively. (**B**) Detection of CD3+Tax+ (red) and CD3+Tax− (blue) cells within the CD4+ cell population, indicating downregulation of CD3 expression in Tax-positive cells. (**C**) Mean fluorescence intensity (MFI) of CD3 in CD4+Tax+ and CD4+Tax− cells, demonstrating a significant reduction in CD3 expression in CD4+Tax+ cells. (**D**) Representative flow cytometry plots of TCRα expression in CD4+ cells from a healthy control and a HAM patient using the anti-TCRα antibody. (**E**) TCRα expression in CD4+ cells of a HAM patient. Tax+ and Tax− cells are depicted in red and blue, respectively, with numbers indicating TCRα positivity in each group. On average, 49.07 ± 14.25% of CD4+Tax− cells and 4.79 ± 4.13% of CD4+Tax+ cells are TCRα-positive in nine HAM patients. (**F**) Representative TCRαβ expression in CD4+ HTLV-1-infected cells using the anti-TCRαβ antibody. Tax+ and Tax− cells are indicated in red and blue, respectively. (**G**) Detection of TCRαβ+Tax+ (red) and TCRαβ+Tax− (blue) cells within the CD4+ population, indicating downregulation of TCRαβ in Tax-positive cells. (**H**) MFI of TCRαβ in CD4+Tax+ and CD4+Tax− cells, showing significantly reduced TCRαβ expression in CD4+Tax+ cells.

Flow cytometric analysis distinguished HTLV-1-infected (Tax-positive) from uninfected (Tax-negative) CD4+ T cells in HAM patients, represented by red and blue populations, respectively (Figure 2A). HTLV-1-infected cells demonstrated substantial downregulation of CD3 expression, while CD4 expression remained comparable to uninfected cells (Figure 2A). Within the CD3+CD4+ population, CD4+Tax+ cells exhibited significantly lower CD3 expression than CD4+Tax− cells (*p* = 0.027, Figure 2B,C), as confirmed by mean fluorescence intensity (MFI) analysis. Expression of TCRα was also markedly reduced in a HAM patient, with 45.70% of CD4+ cells expressing TCRα compared to 72.99% in a healthy control (HC) (Figure 2D). In HTLV-1-infected cells from a HAM patient, only 3.13% of CD4+Tax+ cells expressed TCRα, compared to 52.49% of CD4+Tax− cells (Figure 2E). In 9 HAM patients, the proportion of TCRα+ cells in CD4+Tax+ and CD4+Tax− populations was 4.79 ± 4.13% and 49.07 ± 14.25%, respectively. A similar reduction in TCRαβ expression was observed in CD4+Tax+ cells compared to CD4+Tax− cells (Figure 2F,G), as confirmed by MFI analysis (Figure 2H). In contrast, the intracellular TCRζ component did not show reduced expression (Supplemental Figure S3). Despite diminished CD3 expression, other T cell markers, such as CD2 and CD4, were expressed normally in HTLV-1-infected cells (Supplemental Figure S1), confirming their T cell identity. HTLV-1-infected CD4+ T cells were characterized by a distinct immunophenotype: CD2+CD3^low^TCR^low^CD7^low^CD25^mid^CD27^low^CD28^high^CD45RA-CCR7-CCR4^mid^.

### 2.3. Impaired TCR Signaling in HTLV-1-Infected CD4+ Cells of HAM Patients

**Figure 3:**
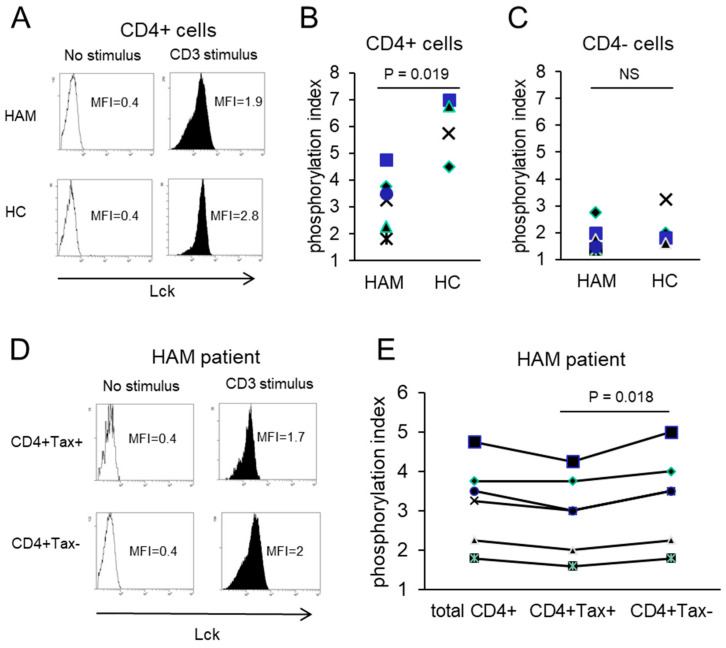
Impaired Lck phosphorylation in HTLV-1-infected CD4+ cells of HAM patients. (**A**) Representative flow cytometry plots of Lck phosphorylation in CD4+ cells from a HAM patient and a healthy control (HC) following CD3 stimulation. The phosphorylation indexes, calculated as MFI with stimulation divided by MFI without stimulation, are 4.75 in the HAM patient and 7.00 in the HC. (**B**) Phosphorylation indexes of CD4+ cells, showing a significant reduction in HAM patients compared to HCs (3.2 vs. 6.0 on average). (**C**) Phosphorylation indexes of CD4- cells are comparable between the two groups, with values of 1.8 in HAM patients and 2.2 in HCs. (**D**) Representative flow cytometry plots of Lck phosphorylation in CD4+Tax+ and CD4+Tax− cells from a HAM patient. The phosphorylation indexes are 4.25 and 5.00, respectively. (**E**) Phosphorylation indexes of Lck in CD4+Tax+ and CD4+Tax− cells from HAM patients, showing a lower index in Tax+ cells (2.93 on average) compared to Tax− cells (3.34).

**Figure 4:**
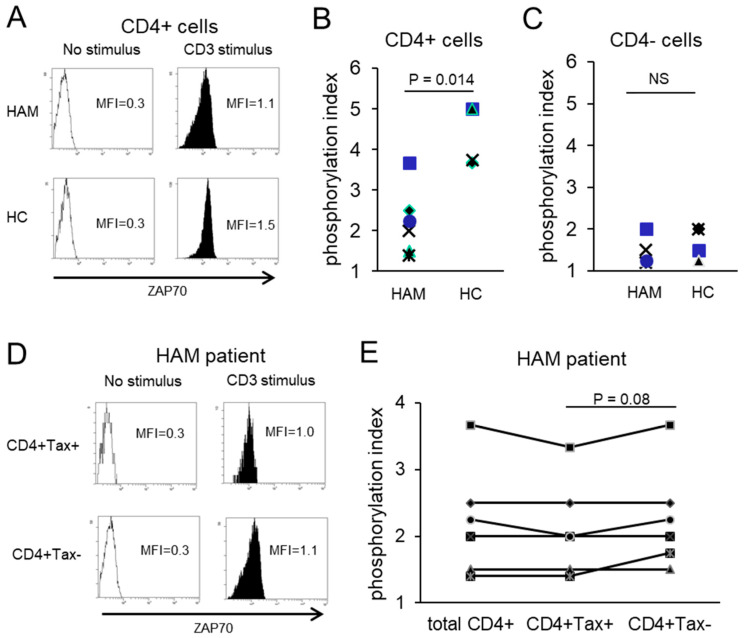
Impaired ZAP70 phosphorylation in HTLV-1-infected CD4+ cells of HAM patients. (**A**) Representative flow cytometry plots of ZAP70 phosphorylation in CD4+ cells from a HAM patient and a healthy control (HC) following CD3 stimulation. The phosphorylation indexes are 3.67 in the HAM patient and 5.00 in the HC. (**B**) Phosphorylation indexes of CD4+ cells, showing a significant reduction in HAM patients compared to HCs (2.22 vs. 4.35 on average). (**C**) Phosphorylation indexes of CD4- cells are comparable between the two groups, with values of 1.53 in HAM patients and 1.69 in HCs. (**D**) Representative flow cytometry plots of ZAP70 phosphorylation in CD4+Tax+ and CD4+Tax− cells from a HAM patient. The phosphorylation indexes were 3.33 and 3.67, respectively. (**E**) Phosphorylation indexes of ZAP70 in CD4+Tax+ and CD4+Tax− cells from HAM patients, showing a lower index in Tax+ cells (2.12 on average) compared to Tax− cells (2.28).

Lck phosphorylation was assessed in CD4+ cells from HAM patients and HCs following CD3 stimulation. The phosphorylation index (MFI of stimulated cells divided by MFI of unstimulated cells) was significantly reduced in a HAM patient (4.75) compared to an HC (7.00) (Figure 3A). CD4+ cells from HCs (n = 4) had a phosphorylation index of 6.0 ± 1.1, whereas those from HAM patients (n = 6) exhibited a significantly lower index of 3.2 ± 1.1, representing a 46.7% decrease (*p* = 0.019, Figure 3B). In contrast, no significant differences were observed in CD4- cells between HAM patients and HCs (1.8 ± 0.52 vs. 2.2 ± 0.74, respectively) (Figure 3C). Within HAM patients, CD4+ Tax-positive cells displayed significantly lower Lck phosphorylation indexes (2.93 ± 1.0) compared to CD4+ Tax-negative cells (3.34 ± 1.2) (*p* = 0.018, Figure 3D,E). Similarly, ZAP70 phosphorylation was significantly impaired in CD4+ cells from HAM patients (2.22 ± 0.83) compared to HCs (4.35 ± 0.75), representing a 49.0% decrease (*p* = 0.014, Figure 4A,B). In contrast, CD4- cells showed no significant differences between HAM patients and HCs (Figure 4C). Among HAM patients, CD4+ Tax-positive cells exhibited lower ZAP70 phosphorylation indexes (2.12 ± 0.71) compared to CD4+ Tax-negative cells (2.28 ± 0.78) (*p* = 0.08, Figure 4D,E). These results indicate that TCR signaling, mediated by Lck and ZAP70, is impaired in HTLV-1-infected CD4+ cells.

### 2.4. Decreased Cytomegalovirus (CMV)-Specific T Cell Response in HTLV-1-Infected CD4+ Cells of HAM Patients

**Figure 5:**
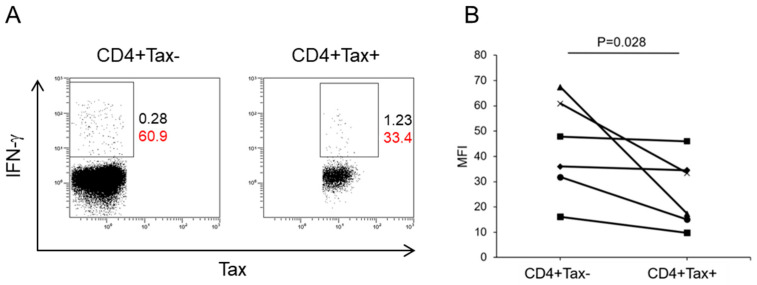
Decreased CMV-specific T cell response in HTLV-1-infected CD4+ cells of HAM patients. (**A**) Flow cytometry plots showing IFN-γ production in CD4+Tax− and CD4+Tax+ cells from a HAM patient following stimulation with CMV antigens. Numbers represent the frequency (black) and MFI (red) of IFN-γ+ cells. (**B**) MFI of IFN-γ is significantly lower in CD4+Tax+ cells (26.0 ± 14.0) compared to CD4+Tax− cells (43.3 ± 19.2).

To assess the antigen-specific response of HTLV-1-infected CD4+ cells to a heterologous virus, PBMCs from HAM patients were cultured with a CMV antigen pool or sterile water, and IFN-γ production was evaluated. In the absence of antigen stimulation, no IFN- γ-positive cells were detected among either CD4+ Tax-positive or Tax-negative populations. Flow cytometric analysis revealed that CD4+ Tax-positive cells exhibited a 40% reduction in IFN-γ MFI (26.0 ± 14.0) compared to CD4+ Tax-negative cells (43.3 ± 19.2) (*p* = 0.028, Figure 5A,B). These results indicate that CD4+ Tax-positive CMV-specific T cells produce lower levels of IFN-γ upon antigen stimulation than CD4+ Tax-negative CMV-specific T cells. These findings suggest that HTLV-1-infected CD4+ cells have a diminished capacity to mount antigen-specific immune responses in HAM patients.

## 3. Discussion

Our study demonstrated that HTLV-1 preferentially targets CD4+ T cells over CD8+ T cells, with significantly reduced expression of the CD3 and TCR complex on CD4+ T cells in HAM patients compared to HCs. Among HTLV-1-infected cells in HAM patients, CD3 and TCR expression were markedly diminished relative to uninfected cells. Furthermore, TCR signaling, as assessed by phosphorylation of Lck and ZAP70 following CD3 stimulation, was significantly impaired in CD4+ T cells from HAM patients compared to those from HCs. This impairment was not observed in CD4-negative cells from either group. Notably, the reduction in TCR signaling was most pronounced in HTLV-1-infected CD4+ T cells compared to their uninfected counterparts. Additionally, HTLV-1-infected, CMV-specific CD4+ T cells exhibited reduced IFN-γ production in response to CMV antigens. These findings suggest that downregulation of the TCR/CD3 complex in HTLV-1-infected CD4+ T cells disrupts TCR signaling and impairs their ability to respond to other viral antigens.

Our findings revealed that a substantial proportion of Tax-positive cells lacked CD3 expression (Figure 2A–C), with over 95% exhibiting diminished TCR levels (Figure 2D–H). Among CD4+ T cells, 5.19% expressed the Tax protein (Figure 1D), and 49.9% of HTLV-1-infected cells expressed Tax protein (Figure 1A), indicating that approximately 10.40% of CD4+ T cells are infected. Notably, total CD4+ T cells from HAM patients exhibited phosphorylation indexes for Lck and ZAP70 that were reduced by 46.7% and 49.0%, respectively, compared to those observed in total CD4+ T cells from HCs (Figure 3B and Figure 4B). These findings indicate a substantial impairment in overall TCR signaling. Furthermore, HTLV-1-infected CD4+ T cells with downregulated TCR/CD3 expression exhibited even greater impairments in TCR signaling and immune responses, including diminished IFN-γ production (Figure 3, Figure 4 and Figure 5). CD4+ T cells play a pivotal role in immune coordination by signaling other immune cells, such as macrophages, CTLs, and B cells, through the secretion of cytokines such as IFN-γ and interleukin-4. Infections like HIV-1, which deplete CD4+ T cells, lead to profound immunodeficiency, leaving the host vulnerable to opportunistic infections and malignancies. Similarly, the significant immune dysfunction observed in HTLV-1-infected CD4+ T cells likely contributes to immunocompromise in individuals with HAM. We demonstrated impaired TCR signaling involving Lck and ZAP70. However, a previous study reported increased mRNA expression of CD3-epsilon, Lck, and ZAP70 in CD4+ T cells from HAM patients compared to HTLV-1 asymptomatic carriers [23]. We did not assess the expression levels of these genes in this study. Several potential explanations for this discrepancy should be considered: (1) the previous study analyzed a mixed population of HTLV-1-infected and uninfected CD4+ T cells, with the latter potentially contributing to increased gene expression; (2) although mRNA levels of Lck and ZAP70 may be elevated in HTLV-1-infected cells, protein levels might be reduced due to unknown epigenetic regulation; or (3) protein levels may be increased, but the signaling pathway could be disrupted by an unidentified mechanism. Further studies are required to resolve this discrepancy.

TCR/CD3 signaling, a pivotal mechanism in immune regulation, is frequently targeted by viral immune evasion strategies. CD3 downregulation has been reported in several viral infections, such as HIV-1 [24,25]. In ATL, CD3 downregulation has been observed, with some ATL cells exhibiting increased CD3 expression upon stimulation with OKT3 monoclonal antibodies, indicating functional suppression of CD3 [22,26]. However, it remains unclear whether similar reductions occur in non-malignant HTLV-1-infected cells in HAM patients. Our study demonstrated decreased CD3 and TCR surface expression in HTLV-1-infected cells, while other T-cell markers such as CD2 and CD4 remained unaffected. Studies of HTLV-1-infected cell lines have revealed suppressed expression of CD3-gamma, -delta, -epsilon, and -zeta genes, resulting in the retention of TCRα and β proteins within the intracellular compartment [27]. This aligns with findings in HIV-1, where CD3 downregulation correlates with defective transcription of the CD3-gamma chain gene [28]. Kinetic studies in HTLV-1 infection suggest a sequential reduction in CD3-gamma mRNA followed by other CD3 subunits, mediated by epigenetic modifications that render their promoters inaccessible [29,30]. Furthermore, transfection assays using HTLV-1 Tax have demonstrated the downregulation of TCR alpha gene transcription [31]. Our findings confirmed that TCR/CD3 complex downregulation in HTLV-1-infected cells impairs TCR signaling, disrupting immune responses such as CMV antigen recognition. This likely diminishes the capacity of HTLV-1-infected CD4+ helper T cells to coordinate immune defense against other pathogens, facilitating immune evasion and viral persistence.

Immunosuppression is well-documented in HTLV-1 carriers and ATL patients, predisposing them to opportunistic infections such as fungal infections, pneumocystis pneumonia, and CMV infection [10]. HAM patients often exhibit high titers of anti-HTLV-1 antibodies, elevated levels of HTLV-1-specific CTLs, and increased serum cytokine levels, indicative of heightened immune responses against HTLV-1 infection [6,8]. However, the high frequency of HTLV-1-specific CTLs correlates with elevated proviral loads in HAM patients, suggesting that the increased immune response is driven by the abundance of HTLV-1-infected cells [32]. It remains unclear whether immune dysfunction extends to responses against other pathogens in HAM patients. Several studies have suggested dysregulated immune responses to non-HTLV-1 pathogens in HAM patients. For instance, reduced generation of CTLs against measles, influenza, and mumps viruses has been reported in HAM patients, and these responses were weaker than those observed in multiple sclerosis patients [33]. Similarly, decreased IFN-γ production by CD8+ T cells in response to viral antigens was noted in HAM patients compared to controls [34]. Additionally, HAM patients exhibited reduced skin reactivity to dinitrochlorobenzene (DNCB) and PPD-tuberculin, as well as diminished lymphoproliferative responses to mitogens, such as phytohemagglutinin or pokeweed mitogen, and these findings were associated with rapid clinical deterioration in HAM patients [15,16]. While these observations predominantly focused on CD8+ T cells, recent studies indicate that herpes simplex virus-specific CD4+ T cells downregulate TCR/CD3 complex expression following HTLV-1 infection [35]. Consistent with these findings, our study demonstrated significantly reduced responses to CD3 stimulation in CD4+ T cells from HAM patients, with particularly profound dysfunction observed in HTLV-1-infected CD4+ T cells. Moreover, we demonstrated that HTLV-1-infected CD4+ T cells exhibited a notably weak immune response to CMV antigens. While opportunistic infections have been documented in HTLV-1 carriers and ATL patients, data on their occurrence in HAM patients remain scarce. Notably, Strongyloides stercoralis infections have been reported in some HAM patients, raising the possibility of increased susceptibility to opportunistic pathogens [36]. Further research is needed to clarify whether HAM patients are predisposed to such infections.

In CD4+ cells, more than 50% of CD4+ cells from HAM patients lacked TCRα expression (Figure 2D). On average, only 4.79% of CD4+ Tax+ cells expressed TCRα, compared to 49.07% in CD4+Tax− cells (Figure 2E). The latter was even lower than that observed in HCs (72.99%) (Figure 2D). The reduced TCR expression in Tax- cells from HAM patients may be attributed to incomplete detection of HTLV-1-infected cells by the Lt-4 antibody or diminished TCR expression in uninfected cells through alternative mechanisms. A time-course study revealed that 49.9% of HTLV-1-infected cells detected via quantitative PCR were positive for intracellular Tax staining (Figure 1A), favoring the former explanation. Further research is required to investigate these possibilities.

Our study also found that HTLV-1-infected CD4+ cells exhibited retained CD2 positivity, and predominantly displayed an effector memory T cell phenotype, as indicated by the absence of CD45RA and CCR7. Additionally, 40.6% of CD4+Tax+ cells expressed CCR4, compared to 20.3% of CD4+Tax− cells (*p* = 0.003, chi-square test; odds ratio: 2.68) (Supplemental Figure S1). These findings suggest that CCR4 is preferentially expressed on HTLV-1-infected cells, though it represents only 40.6% of the infected cell population. A recent clinical trial using an anti-CCR4 antibody, mogamulizumab, demonstrated a 64.9% reduction in proviral load among HAM patients [37], highlighting its efficacy against CCR4+ HTLV-1-infected cells. However, the persistence of CCR4-negative infected cells underscores the need for alternative surface markers with high sensitivity and specificity for HTLV-1-infected cells to target the entire infected cell population effectively.

In conclusion, our study demonstrates that downregulation of the TCR/CD3 complex in HTLV-1-infected CD4+ T cells significantly impairs TCR signaling, contributing to immune dysfunction in HAM patients. These findings emphasize the profound impact of HTLV-1 infection on cellular immune responses and its potential role in disease progression, underscoring the necessity for novel therapeutic approaches targeting all HTLV-1-infected cells.

## 4. Materials and Methods

### 4.1. Subjects

Peripheral blood samples were collected from nine patients with HAM and four HCs after obtaining informed consent. The diagnosis of HAM was confirmed based on the WHO criteria. PBMCs were isolated using standard density gradient centrifugation and cryopreserved in liquid nitrogen until use. The study was approved by the Ethics Committee of Kagoshima University and conducted in accordance with the Declaration of Helsinki.

### 4.2. Cell Culture

Cryopreserved PBMCs were thawed, washed, and cultured in RPMI 1640 medium supplemented with 10% fetal bovine serum, 100 U/mL penicillin, and 100 μg/mL streptomycin. To detect HTLV-1-infected cells, PBMCs were incubated with brefeldin A (10 μg/mL; Sigma-Aldrich, Tokyo, Japan), a protein transport inhibitor, for 8 h at 37 °C. For phosphorylation assays of Lck and ZAP70, PBMCs were stimulated with 1 μg/mL of anti-CD3 epsilon monoclonal antibody (OKT3; eBioscience, Waltham, MA, USA) for 20 min at 4 °C, followed by washing in cold PBS containing 10% FBS. Secondary anti-mouse IgG antibody was then applied to facilitate capping, with subsequent incubation for 20 min at 4 °C. The cells were transferred to a pre-warmed 96-well V-bottom culture plate, and phosphorylation was terminated by the addition of H_2_O_2_ for 3 min at room temperature (RT). This was followed by fixation with 1.5% paraformaldehyde for 10 min at RT. After washing, the cells were permeabilized with cold methanol for 30 min at 4 °C. Subsequently, the cells were stained with Lt-4 antibody and antibodies targeting phosphorylated Lck or ZAP70 [38]. To detect CMV-specific T cells, PBMCs from HAM patients were incubated with a CMV antigen pool (PepTivator CMV pp65; Miltenyi Biotec, Tokyo, Japan) in the presence of brefeldin A [39]. The MFI of stimulated and unstimulated cells was measured, and the phosphorylation index was calculated using the following formula: (phosphorylation index) = (MFI of stimulated cells)/(MFI of unstimulated cells).

### 4.3. Flow Cytometry

Surface markers, including CD3, CD4, CD8, and TCR, were stained with the appropriate antibodies for 15 min at RT. After staining, cells were fixed in 4% paraformaldehyde and permeabilized with a staining buffer containing 0.1% saponin for subsequent intracellular staining. Intracellular Tax protein was detected by incubating cells with the Lt-4 mouse IgG3 antibody (kindly provided by Dr. Tanaka, University of the Ryukyus) for 15 min at RT, followed by secondary staining with a PE-conjugated anti-mouse IgG3 antibody (SouthernBiotech, Birmingham, AL, USA) for 15 min at RT. For simultaneous detection of intracellular Tax and phosphorylated molecules such as Lck or ZAP70, cells were fixed and stained sequentially with the Lt-4 antibody and an antibody specific to the phosphorylated molecule, followed by staining with the secondary anti-mouse IgG3 antibody, anti-CD4, and anti-CD3 antibodies. CMV-specific CD4+ T cells were identified via intracellular IFN-γ staining, using an anti-IFN-γ antibody, and evaluated based on MFI. Intracellular FoxP3 staining was performed according to the manufacturer’s instructions. For detailed analyses of HTLV-1-infected cells, samples containing a minimum of 50 Tax-positive cells were included. Antibodies used targeted CD4, CD8, CD25, CD27, CD28, CD45RA, TCRζ (Beckman Coulter, Tokyo, Japan), CD2, CD3, CD7, CD26, TCRα (clone T10B9.1A-31), TCRαβ (clone WT31), CCR4, IFN-γ, phosphorylated Lck, phosphorylated ZAP70 (Becton Dickinson, Franklin Lakes, NJ, USA), CCR7 (R&D Systems, Minneapolis, MN, USA), and FoxP3 (eBioscience, Waltham, MA, USA). Isotype controls were appropriately utilized. Fluorescence signals were acquired using an Epics XL flow cytometer, and data were analyzed with Expo32 software (Beckman Coulter, Tokyo, Japan). For the data presented in Figure 2F–H, signals were acquired using a CytoFLEX flow cytometer and analyzed with CytExpert software ver. 2.4 (Beckman Coulter, Tokyo, Japan). Lymphocyte populations were gated based on forward- and side-scatter profiles.

### 4.4. Quantification of HTLV-1 Proviral Load (PVL)

DNA was extracted from PBMCs using the DNAeasy Blood & Tissue Kit (QIAGEN, Hilden, Germany). Quantitative real-time PCR was conducted using a TaqMan-based assay, as described previously [7]. Briefly, 10 ng of DNA and a standard template were subjected to 30 PCR cycles with HTLV-1 Tax primers, β-actin primers, and TaqMan probes (Applied Biosystems, Tokyo, Japan) using a OneStep Plus real-time PCR system (Thermo Fisher Scientific, Waltham, MA, USA). All reactions were performed in triplicate. The copy number of HTLV-1 Tax and β-actin genes was calculated using standard curves. The PVL was expressed as copies per 100 cells, calculated as: PVL (copies/100 cells) = (HTLV-1 tax copy number)/(1/2 × β-actin copy number) × 100.

### 4.5. Statistical Analysis

Statistical comparisons were performed using the two-tailed Mann–Whitney U test for unpaired data and the Wilcoxon signed-rank test for non-parametric paired data. Chi-square tests were applied for 2 × 2 analyses. A *p*-value of <0.05 was considered statistically significant.

## Data Availability

The data presented in this study are available on request to the corresponding author.

## Supplementary Materials

The following supporting information can be downloaded at: https://www.mdpi.com/article/10.3390/ijms26041706/s1.

## References

[1] Richardson J.H., Edwards A.J., Cruickshank J.K., Rudge P., Dalgleish A.G. (1990). In vivo cellular tropism of human T-cell leukemia virus type 1. J. Virol..

[2] Gessain A., Cassar O. (2012). Epidemiological Aspects and World Distribution of HTLV-1 Infection. Front. Microbiol..

[3] Uchiyama T., Yodoi J., Sagawa K., Takatsuki K., Uchino H. (1977). Adult T-cell leukemia: Clinical and hematologic features of 16 cases. Blood.

[4] Osame M., Usuku K., Izumo S., Ijichi N., Amitani H., Igata A., Matsumoto M., Tara M. (1986). HTLV-I associated myelopathy, a new clinical entity. Lancet.

[5] Gessain A., Barin F., Vernant J.C., Gout O., Maurs L., Calender A., de The G. (1985). Antibodies to human T-lymphotropic virus type-I in patients with tropical spastic paraparesis. Lancet.

[6] Osame M., Matsumoto M., Usuku K., Izumo S., Ijichi N., Amitani H., Tara M., Igata A. (1987). Chronic progressive myelopathy associated with elevated antibodies to human T-lymphotropic virus type I and adult T-cell leukemialike cells. Ann. Neurol..

[7] Nagai M., Usuku K., Matsumoto W., Kodama D., Takenouchi N., Moritoyo T., Hashiguchi S., Ichinose M., Bangham C.R., Izumo S. (1998). Analysis of HTLV-I proviral load in 202 HAM/TSP patients and 243 asymptomatic HTLV-I carriers: High proviral load strongly predisposes to HAM/TSP. J. Neurovirol..

[8] Jacobson S., Shida H., McFarlin D.E., Fauci A.S., Koenig S. (1990). Circulating CD8+ cytotoxic T lymphocytes specific for HTLV-I pX in patients with HTLV-I associated neurological disease. Nature.

[9] Kubota R., Furukawa Y., Izumo S., Usuku K., Osame M. (2003). Degenerate specificity of HTLV-1-specific CD8+ T cells during viral replication in patients with HTLV-1-associated myelopathy (HAM/TSP). Blood.

[10] Kawano N., Nagahiro Y., Yoshida S., Tahara Y., Himeji D., Kuriyama T., Tochigi T., Nakaike T., Shimokawa T., Yamashita K. (2019). Clinical features and treatment outcomes of opportunistic infections among human T-lymphotrophic virus type 1 (HTLV-1) carriers and patients with adult T-cell leukemia-lymphoma (ATL) at a single institution from 2006 to 2016. J. Clin. Exp. Hematop..

[11] Maeda T., Babazono A., Nishi T., Yasui M., Matsuda S., Fushimi K., Fujimori K. (2015). The Impact of Opportunistic Infections on Clinical Outcome and Healthcare Resource Uses for Adult T Cell Leukaemia. PLoS ONE.

[12] Marsh B.J. (1996). Infectious complications of human T cell leukemia/lymphoma virus type I infection. Clin. Infect. Dis..

[13] Mascarenhas R.E., Brodskyn C., Barbosa G., Clarencio J., Andrade-Filho A.S., Figueiroa F., Galvao-Castro B., Grassi F. (2006). Peripheral blood mononuclear cells from individuals infected with human T-cell lymphotropic virus type 1 have a reduced capacity to respond to recall antigens. Clin. Vaccine Immunol..

[14] Rosadas C., Taylor G.P. (2022). HTLV-1 and Co-infections. Front. Med..

[15] Kuroda Y., Takashima H. (1990). Impairment of cell-mediated immune responses in HTLV-I-associated myelopathy. J. Neurol. Sci..

[16] Kuroda Y., Fujiyama F., Nagumo F. (1991). Analysis of factors of relevance to rapid clinical progression in HTLV-I-associated myelopathy. J. Neurol. Sci..

[17] Werlen G., Palmer E. (2002). The T-cell receptor signalosome: A dynamic structure with expanding complexity. Curr. Opin. Immunol..

[18] Germain R.N. (2001). The T cell receptor for antigen: Signaling and ligand discrimination. J. Biol. Chem..

[19] Gascoigne N.R., Casas J., Brzostek J., Rybakin V. (2011). Initiation of TCR phosphorylation and signal transduction. Front. Immunol..

[20] Buchacz K., Lau B., Jing Y., Bosch R., Abraham A.G., Gill M.J., Silverberg M.J., Goedert J.J., Sterling T.R., Althoff K.N. (2016). Incidence of AIDS-Defining Opportunistic Infections in a Multicohort Analysis of HIV-infected Persons in the United States and Canada, 2000–2010. J. Infect. Dis..

[21] Mohanty S., Harhaj E.W. (2020). Mechanisms of Oncogenesis by HTLV-1 Tax. Pathogens.

[22] Suzushima H., Hattori T., Asou N., Wang J.X., Nishikawa K., Okubo T., Anderson P., Takatsuki K. (1991). Discordant gene and surface expression of the T-cell receptor/CD3 complex in adult T-cell leukemia cells. Cancer Res..

[23] Pinto M.T., Malta T.M., Rpdrigues E.S., Takayanagi O.M., Tanaka Y., Covas D.T., Kashima S. (2015). T cell receptor signaling pathway is overexpressed in CD4(+) T cells from HAM/TSP individuals. Braz. J. Infect. Dis..

[24] Linette G.P., Hartzman R.J., Ledbetter J.A., June C.H. (1988). HIV-1-infected T cells show a selective signaling defect after perturbation of CD3/antigen receptor. Science.

[25] Mesner D., Hotter D., Kirchhoff F., Jolly C. (2020). Loss of Nef-mediated CD3 down-regulation in the HIV-1 lineage increases viral infectivity and spread. Proc. Natl. Acad. Sci. USA.

[26] Matsuoka M., Hattori T., Chosa T., Tsuda H., Kuwata S., Yoshida M., Uchiyama T., Takatsuki K. (1986). T3 surface molecules on adult T cell leukemia cells are modulated in vivo. Blood.

[27] de Waal Malefyt R., Yssel H., Spits H., de Vries J.E., Sancho J., Terhorst C., Alarcon B. (1990). Human T cell leukemia virus type I prevents cell surface expression of the T cell receptor through down-regulation of the CD3-gamma, -delta, -epsilon, and -zeta genes. J. Immunol..

[28] Willard-Gallo K.E., Van de Keere F., Kettmann R. (1990). A specific defect in CD3 gamma-chain gene transcription results in loss of T-cell receptor/CD3 expression late after human immunodeficiency virus infection of a CD4+ T-cell line. Proc. Natl. Acad. Sci. USA.

[29] Akl H., Badran B., Dobirta G., Manfouo-Foutsop G., Moschitta M., Merimi M., Burny A., Martiat P., Willard-Gallo K.E. (2007). Progressive loss of CD3 expression after HTLV-I infection results from chromatin remodeling affecting all the CD3 genes and persists despite early viral genes silencing. Virol. J..

[30] Yssel H., de Waal Malefyt R., Duc Dodon M.D., Blanchard D., Gazzolo L., de Vries J.E., Spits H. (1989). Human T cell leukemia/lymphoma virus type I infection of a CD4+ proliferative/cytotoxic T cell clone progresses in at least two distinct phases based on changes in function and phenotype of the infected cells. J. Immunol..

[31] Wencker M., Sausse C., Derse D., Gazzolo L., Duc Dodon M. (2007). Human T-cell leukemia virus type 1 Tax protein down-regulates pre-T-cell receptor alpha gene transcription in human immature thymocytes. J. Virol..

[32] Kubota R., Kawanishi T., Matsubara H., Manns A., Jacobson S. (2000). HTLV-I specific IFN-gamma+ CD8+ lymphocytes correlate with the proviral load in peripheral blood of infected individuals. J. Neuroimmunol..

[33] Jacobson S., Gupta A., Mattson D., Mingioli E., McFarlin D.E. (1990). Immunological studies in tropical spastic paraparesis. Ann. Neurol..

[34] Manuel S.L., Sehgal M., Connolly J., Makedonas G., Khan Z.K., Gardner J., Betts M.R., Jain P. (2013). Lack of recall response to Tax in ATL and HAM/TSP patients but not in asymptomatic carriers of human T-cell leukemia virus type 1. J. Clin. Immunol..

[35] Inatsuki A., Yasukawa M., Kobayashi Y. (1989). Functional alterations of herpes simplex virus-specific CD4+ multifunctional T cell clones following infection with human T lymphotropic virus type I. J. Immunol..

[36] Maciel E., Espinheira L., Brites C. (1999). Strongyloidiasis as an Opportunistic Infection in a HAM/TSP Patient. Braz. J. Infect. Dis..

[37] Sato T., Coler-Reilly AL G., Yagishita N., Araya N., Inoue E., Furuta R., Watanabe T., Uchimaru K., Matsuoka M., Matsumoto N. (2018). Mogamulizumab (Anti-CCR4) in HTLV-1-Associated Myelopathy. N. Engl. J. Med..

[38] Krutzik P.O., Nolan G.P. (2003). Intracellular phospho-protein staining techniques for flow cytometry: Monitoring single cell signaling events. Cytom. A.

[39] Peggs K.S., Thomson K., Samuel E., Dyer G., Armoogum J., Chakraverty R., Pang K., Mackinnon S., Lowdell M.W. (2011). Directly selected cytomegalovirus-reactive donor T cells confer rapid and safe systemic reconstitution of virus-specific immunity following stem cell transplantation. Clin. Infect. Dis..

