# Supplementary figures and images for: T-Cell Receptor/CD3 Downregulation and Impaired Signaling in HTLV-1-Infected CD4+ T Cells of HAM Patients
Source: Int J Mol Sci. 2025 Feb 17;26(4):1706. doi: 10.3390/ijms26041706 (PMC11855110; doi:10.3390/ijms26041706)

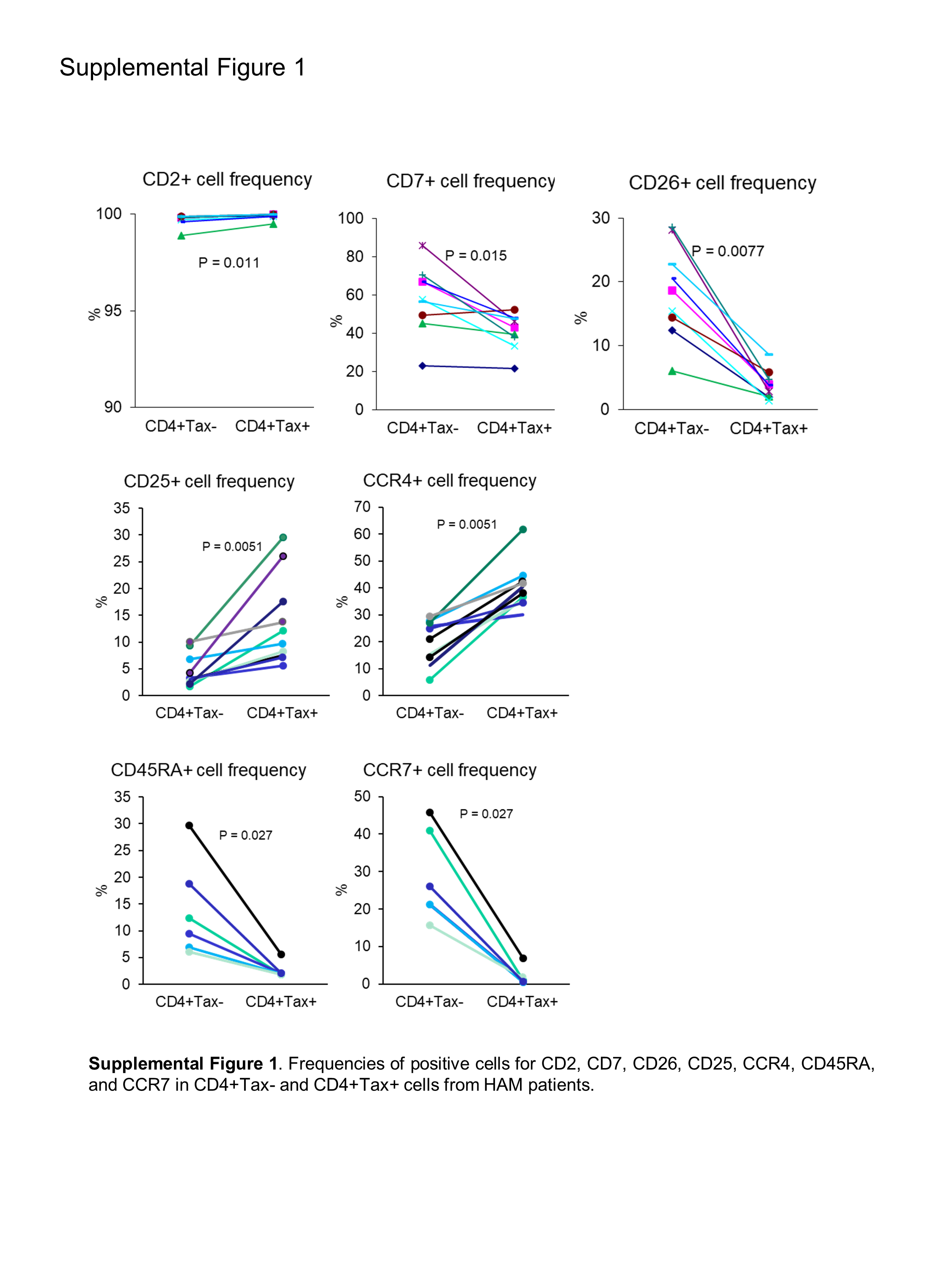

Supplement: Supplementary file 1 [file ijms-26-01706-s001.zip › SupF1.TIF]

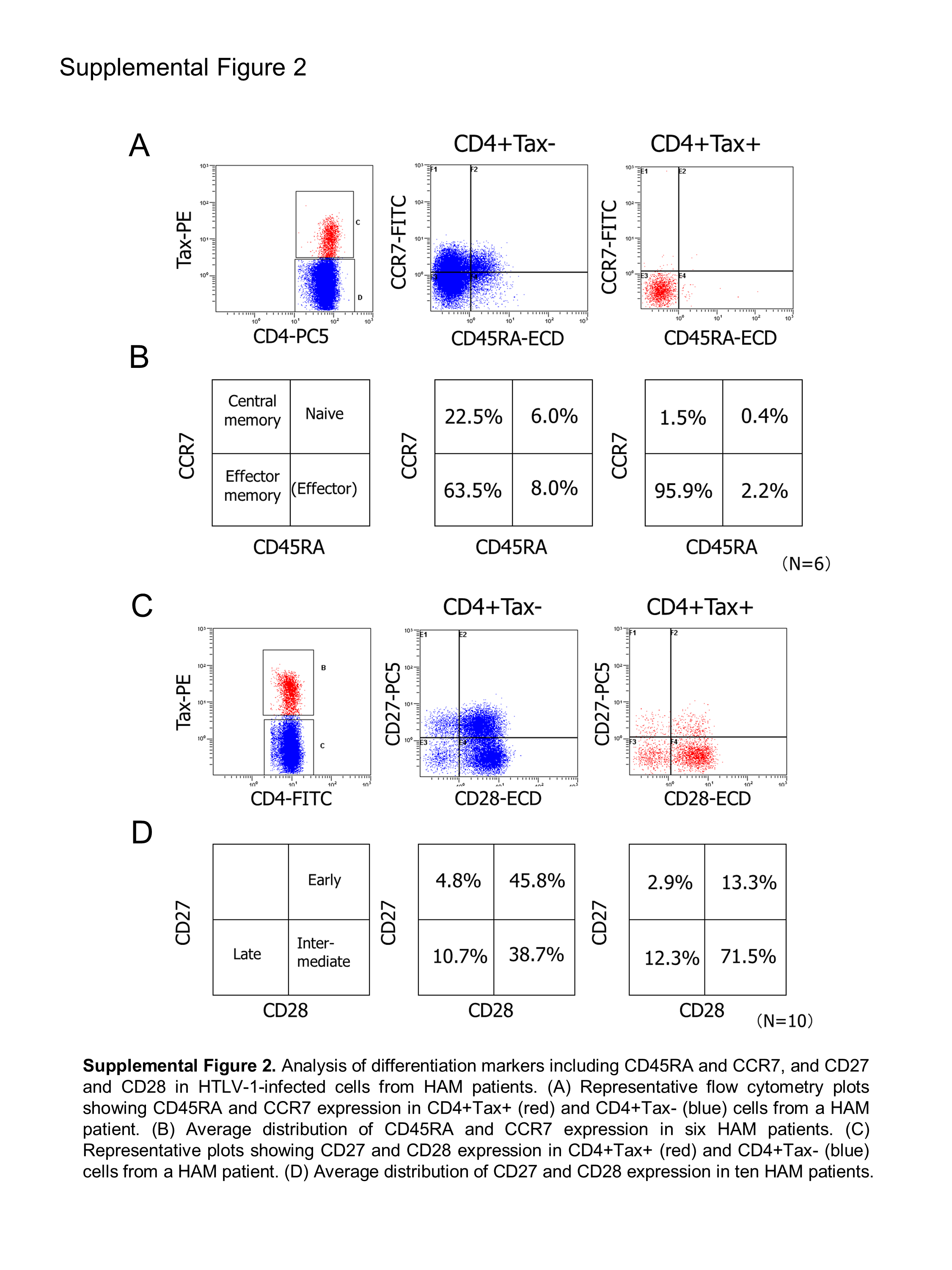

Supplement: Supplementary file 1 [file ijms-26-01706-s001.zip › SupF2.TIF]

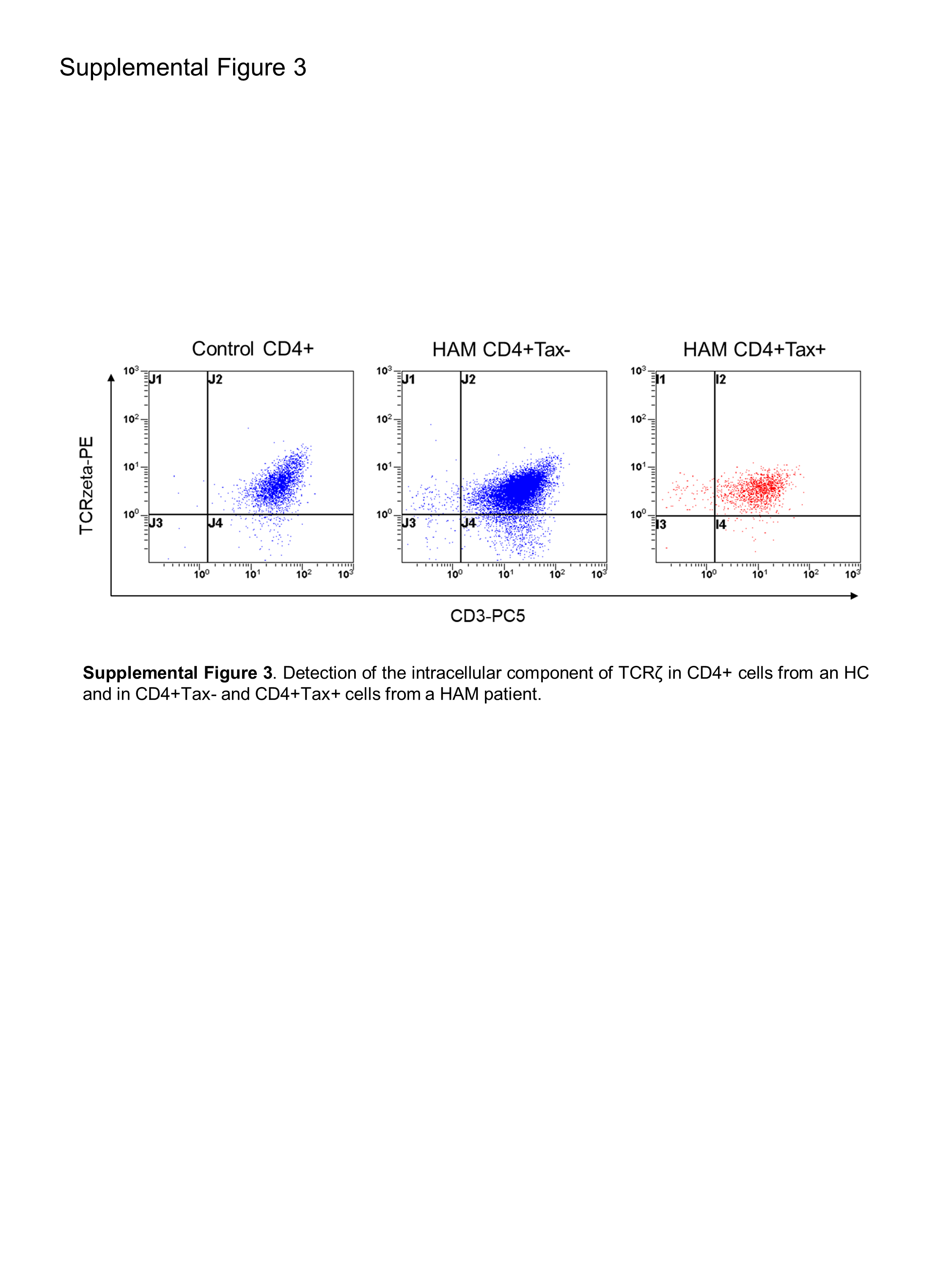

Supplement: Supplementary file 1 [file ijms-26-01706-s001.zip › SupF3.TIF]
